# Draft Genome Sequence of *Mycobacterium interjectum* Strain ATCC 51457^T^

**DOI:** 10.1128/genomeA.00452-16

**Published:** 2016-05-26

**Authors:** Anthony Levasseur, Shady Asmar, Catherine Robert, Michel Drancourt

**Affiliations:** Aix-Marseille Université, URMITE, CNRS, UMR 7278, IRD 198, Faculté de Médecine, Marseille, France

## Abstract

*Mycobacterium interjectum* is a nontuberculosis species rarely responsible for human infection. The draft genome of *M. interjectum* ATCC 51457^T^ comprises 5,927,979 bp, exhibiting 67.91% G+C content, 5,314 protein-coding genes, and 51 predicted RNA genes.

## GENOME ANNOUNCEMENT

*Mycobacterium interjectum* was delineated as a new species of nontuberculous mycobacteria most closely related to *Mycobacterium simiae* (1, 2), *Mycobacterium saskatchewanense* sp. nov. (3), and *Mycobacterium paraense* sp. nov. (4). *M. interjectum* is an opportunistic pathogen mainly isolated from diseased lymph nodes (1, 5–8) and the respiratory tract (5, 9–11). A few other cases have caused meningoencephalitis (12) and cutaneous infection (13). Also, *M. interjectum* has been isolated from mammals (14–16), fish (17, 18), and clear water (19, 20). It is therefore of medical and general interest to further describe the genome of this species, and we performed whole-genome sequencing of the *M. interjectum* ATCC 51457^T^ strain. Genomic DNA isolated from *M. interjectum* grown in MGIT Middlebrook liquid culture (Becton, Dickinson, Le Pont-de-Claix, France) was sequenced in two Illumina MiSeq runs (Illumina, Inc., San Diego, CA) using a 5.9-kb mate-paired library. Reads were trimmed using Trimmomatic (21) and assembled using Velvet (version 1.2.03) (22). Contigs were combined by SSPACE version 2 (23), Opera version 2 (24) helped by GapFiller version 1.10 (25), and homemade tools in Python to refine the set. The *M. interjectum* strain ATCC 51457^T^ draft genome consists of 30 scaffolds and 221 contigs containing 5,927,979 bp and 67.91% G+C content. Noncoding genes and miscellaneous features were predicted using RNAmmer (26), ARAGORN (27), Rfam (28), PFAM (29), and Infernal (30). Coding DNA sequences (CDSs) were predicted using Prodigal (31), and functional annotation was achieved using BLAST+ (32) and HMMER3 (33) against the UniProtKB database (34). The genome was shown to encode at least 51 predicted RNAs, including three rRNAs and 48 tRNAs. A total of 5,314 identified genes yielded a coding capacity of 4,706,163 bp (coding percentage, 79.3%). Among these genes, 4,431 (83.38%) were found to be putative proteins, and 883 (16.6%) were assigned as hypothetical proteins. Moreover, 3,056 genes matched a least one sequence in the Clusters of Orthologous Groups database (35, 36) with BLASTP default parameters. *In silico* DNA-DNA hybridization (DDH) (37) was performed with 16 reference genomes selected on the basis of their 16S rRNA gene proximity with *M. interjectum*. The *M. interjectum* genome was locally aligned 2-by-2 using BLAT algorithm (38, 39) against each one of the 16 selected genomes, and DDH values were estimated from a generalized linear model (40). The DDH was 27.8% (±2.43%) for *Mycobacterium smegmatis* mc^2^155, 26.70% (±2.42%) for *Mycobacterium ulcerans* Agy99, 25.9% (±2.41%) for *Mycobacterium avium* subsp. *paratuberculosis* K-10, *Mycobacterium intracellulare* ATCC 13950, and *Mycobacterium indicus pranii* MTCC 9506, 23.6% (±2.38%) for *Mycobacterium kansasii* ATCC 12478, 23.5% (±2.38%) for *Mycobacterium tuberculosis* H37Rv and *Mycobacterium bovis* AF2122/97, 22.4% (±2.36%) for *Mycobacterium marinum* M and *Mycobacterium liflandii* 128FXT, 20.9% (±2.33%) for *Mycobacterium chubuense* NBB4, 20.8% (±2.33%) for *Mycobacterium vanbaalenii* PYR-1, 20.5% (±2.32%) for *Mycobacterium gilvum* PYR-GCK, 20.4% (±2.32%) for *Mycobacterium leprae* TN and *Mycobacterium neoaurum* VKM Ac-1815D, and 20.2% (±2.31%) for *Mycobacterium rhodesiae* NBB3.

### Nucleotide sequence accession number.

The *M. interjectum* strain ATCC 51457^T^ genome sequence has been deposited at EMBL under the accession no. FJVQ00000000. The version described in this paper is the first version.
